# Supplementary material for: WordCommentsAnalyzer: A windows software tool for qualitative research
Source: F1000Res. 2018 Sep 4;7:536. Originally published 2018 May 3. [Version 2] doi: 10.12688/f1000research.14819.2 (PMC6137411; doi:10.12688/f1000research.14819.2)
Supplement: Supplementary file 3 [file f1000research-7-17614-s0002.tgz › b2a79496-4ada-47c4-9682-a99ce867836d.zip!/Eight Iranian Health Journals/Journal of Health System Research _41.docx]

© Journal of Health System Research . This work is licensed under CC BY-NC 4.0 (https://creativecommons.org/licenses/by-nc/4.0/).

Volume & Issue: دوره 7، شماره 4؛ 1390

Title: SHERPA ارزيابي و مديريت خطاهای انسانی در اپراتورهای اتاق کنترل پالایشگاه نفت اصفهان با استفاده از روش

http://hsr.mui.ac.ir/index.php/jhsr/article/view/248

مقدمه: در بسیاری از محیط‌های شغلی با وجود فن آوری‌های حساس و پیچیده، خطاهای انسانی به دلیل منتهی شدن به حوادث ناگوار از اهمیت بالایی برخوردار است. به همین دلیل جهت پیشگیری و محدود ساختن پیامدهای ناشی از خطاهای انسانی، شناسایی و علت‌یابی آن‌ها لازم است. هدف این مطالعه شناسایی، ارزيابي و كنترل خطاهای انسانی در واحد تقطیر پالایشگاه نفت اصفهان است. روش‌ها: این مطالعه در سال 1389 صورت گرفت. جمع‌آوری اطلاعات با استفاده از روش مشاهده وظایف و مصاحبه با مسؤولین ایمنی، سرپرستان واحد و شیفت و اپراتورها انجام شد و 8 وظیفه بحرانی تعیین شد. سپس تجزیه و تحلیل سلسله مراتبی (Hierarchical task analysis، HTA) وظایف بحرانی صورت گرفت. سپس خطاهای انسانی هر یک از وظایف بحرانی با روش SHERPA (Systematic human error reduction prediction approach) شناسایی شد. یافته‌ها: با تجزیه و تحلیل برگه‌های کاری SHERPA، 198 خطای انسانی شناسایی گردید. از این تعداد 134 خطا (64/67 درصد) از نوع عملکردی، 23 خطا (61/11 درصد) چک کردنی، 11 خطا (6/5 درصد) ارتباطی، 24 خطا (12/12 درصد) بازگشتی و 6 خطا (03/3 درصد) انتخابی طبقه‌بندی شد. 64 درصد از خطای انسانی در اپراتور اتاق کنترل از احتمال رخداد "پایین" و 36 درصد احتمال رخداد "متوسط" برآورد شد. همچنین 59 درصد از خطاهای شناسایی شده اپراتور اتاق کنترل، بدون کنترل‌کننده لازم بودند ولی از این میان، تنها 29 درصد پیامد حساس داشتند. نتیجه‌گیری: نتایج این مطالعه نشان داد که روش SHERPA می‌تواند به عنوان یک روش مؤثر جهت شناسایی خطاهای انسانی در اتاق کنترل واحدهای حساس پالایشگاه‌های نفت مورد استفاده قرار گیرد.

Title: تعیین الگوی عوامل بازدارنده از مشارکت در فعالیت‌های ورزشی کارکنان بیمارستان‌های آموزشی شهر اصفهان بر اساس مدل سلسله مراتبی اوقات فراغت

http://hsr.mui.ac.ir/index.php/jhsr/article/view/249

مقدمه: رابطه بین اوقات فراغت و کار از دیرباز مورد توجه است. از آن به عنوان عاملی در جهت ایجاد انگیزه شغلی و رضایتمندی در کارکنان یاد می‌شود. در این راستا نیاز کارکنان در محیط‌های کاری ویژه مثل بیمارستان‌ها به خاطر وجود تنیدگی شغلی و فشارهای کاری، بیش از بیش احساس می‌شود. از این رو توجه به اوقات فراغت و برنامه‌ریزی دقیق آن با تأکید بر فعالیت‌های جسمانی، عاملی در جهت شادابی روحی و آمادگی جسمانی کارکنان است. این امر زمینه را برای ارایه خدمات بهتر از سوی بیمارستان‌ها فراهم می‌کند. هدف از پژوهش حاضر، تعیین الگوی عوامل بازدارنده از مشارکت در فعاليت‌های ورزشی در اوقات فراغت کارکنان بيمارستان‌های آموزشی شهر اصفهان با استفاده از مدل سلسله مراتبی می‌باشد. روش‌ها پژوهش حاضر از دسته مطالعات توصیفی است که به صورت مقطعی و پیمایشی اجرا شد. جامعه آماري در اين پژوهش، کليه کارکنان شاغل در بخش‌هاي مختلف بيمارستان‌هاي آموزشي تحت پوشش دانشگاه علوم پزشکي اصفهان بود. نمونه مورد مطالعه از طريق روش نمونه‌گيري خوشه‌اي چند مرحله‌اي همراه با طبقه‌بندي متناسب با حجم طبقات انتخاب شد. کل حجم نمونه مورد نياز براي انجام اين پژوهش بر اساس روش کرجسي و مورگان 345 نفر تعيين گرديد. اطلاعات مورد نياز از طريق پرسش‌نامه‌اي که در اختيار شرکت‌کنندگان قرار گرفت، جمع‌آوري گردید. در اين پژوهش، تجزيه و تحليل داده‌ها با استفاده از روش‌هاي آمار توصيفي و تحليل عاملي اکتشافی صورت گرفت. یافته‌ها: در این پژوهش، ترتیب و توالی عوامل سه گانه فردی، بین فردی و ساختاری تابع مدل سلسله مراتبی عوامل بازدارنده برای پرداختن به فعالیت‌های ورزشی در اوقات فراغت به دست نیامد. به ترتیب عوامل ساختاری، عوامل فردی و در نهایت عوامل بین فردی به لحاظ اولویت قرار گرفتند. در عوامل ساختاری عوامل‌ اقتصادی بیشترین نقش را در عدم مشارکت در فعالیت‌های ورزشی ایفا نمودند. بحث و نتیجه‌گیری جهت افزایش مشارکت کارکنان بیمارستان‌ها در فعالیت‌های ورزشی در اوقات فراغت، مدیران بیمارستان‌ها می‌بایست علاوه بر تلاش برای تعدیل و به حداقل رساندن عوامل بازدارنده، با فرهنگ‌سازی و ایجاد بستری مناسب برای مشارکت کلیه کارکنان بیمارستان‌ها در فعالیت‌های ورزشی، سلامت جسمانی و روانی کارکنان را بهبود و بهره‌وری آنان را افزایش دهند.

Title: بررسی شیوع خوددرمانی و عوامل قابل تعدیل مؤثر بر آن بر اساس الگوی اعتقاد بهداشتی در سالمندان شهر گناباد

http://hsr.mui.ac.ir/index.php/jhsr/article/view/250

مقدمه: حفظ سلامت از دغدغه‌های دیرین انسان‌ها است و از مؤلفه‌های متعددی تأثیر می‌پذیرد. اعلامیه‌ها و راهبردهای جهانی سلامت همواره بر تهیه و مصرف صحیح داروها و درمان مناسب تأکید می‌نماید. خوددرمانی در ایران از معضلات سلامتی به شمار می‌رود و سه برابر متوسط جهانی و رتبه دوم در آسیا را دارا است. برای اثرگذاری بر این معضل، یکی از روش‌های علمی، بررسی عوامل مؤثر بر این رفتار بر اساس الگوی اعتقاد بهداشتی است. در این پژوهش عوامل مؤثر بر خوددرمانی با تعیین میزان تأثیر مورد شناسایی قرار گرفتند تا جهت اثرگذاری اولویت‌بندی شوند. روش‌کار: دراین پژوهش توصیفی تحلیلی مقطعی، 380 سالمند شهر گناباد به روش نمونه‌گیری طبقه‌ای با تخصیص متناسب انتخاب شدند. اطلاعات بر اساس پرسش‌نامه مشتمل بر 47 سؤال به روش مصاحبه سازمان یافته در بخش‌های الگوی اعتقاد بهداشتی جمع‌آوری گردید. روایی و پایایی به روش‌های علمی تأییدگردید. داده‌ها با نرم‌افزار SPSS تجزیه و تحلیل شد. یافته‌ها: میانگین سنی سالمندان 31/5 ± 2/66 بود. 1/36 درصد بازنشسته، 4/29 درصد تحصیلات ابتدایی و 9/53 درصد بیمه درمانی داشتند. 6/77 درصد خودسرانه دارو مصرف می‌کردند و 3/85 درصد در منزل دارو نگه می‌داشتند. 3/42 درصد داروهای مصرف شده مسکن و 1/15 درصد مربوط به اعصاب و روان بودند. میانگین نمره حساسیت 21/50 و موانع درک‌شده 23/70 بود. بین جنس، وضعیت تأهل و تحصیلات با خوددرمانی ارتباط معنی‌داری وجود داشت. مهم‌ترین علت خوددرمانی، تجربه بهبودی قبلی و بی خطر دانستن داروها ذکر شد. وسایل ارتباط جمعی مؤثرترین راهنمای عمل در پیشگیری از خوددرمانی عنوان شد. نتیجه‌گیری: در این مطالعه شیوع خوددرمانی بالا است و عوامل قابل تعدیل در شرایط نامطلوبی قرار داشتند. افراد در موارد الگوی اعتقاد بهداشتی حتی 50 درصد حداقل‌ها را کسب نکردند ولی نمره Self-efficacy مطلوب به دست آمد که این اطمینان کاذب، خوددرمانی را تشدید کرد.

Title: پاداش درک‌شده رانندگی غیر ایمن و هزینه درک‌شده رانندگی ایمن به عنوان تعیین‌کننده‌های وضعیت رانندگی مردم شهر یزد- کاربردی از تئوری انگیزش محافظت

http://hsr.mui.ac.ir/index.php/jhsr/article/view/251

مقدمه: حوادث ترافیکی یکی از مشکلات بزرگ سلامت عمومی به شمار می‌رود که پیشگیری مداوم و مؤثر از آن‌ها نیازمند تلاش‌های هماهنگ و همه جانبه است. پاداش و هزینه درک‌شده، از سازه‌های تئوری انگیزش محافظت می‌باشند. ماهیت آن‌ها به خوبی تبیین نشده است ولی شواهدی مبنی بر تأثیرگذاری آن‌ها بر وضعیت رانندگی وجود دارد. بنابراین مطالعه حاضر با هدف تعیین وضعیت سازه‌های فوق در زمینه رانندگی انجام شد. روش‌ها: در این مطالعه مقطعی، 379 نفر از افراد 19 ساله و بالاتر مورد مطالعه قرار گرفتند. این افراد دارای گواهینامه رانندگی بودند و به روش خوشه‌ای تصادفی از جمعیت تحت پوشش مراکز بهداشتی درمانی شهری شهر یزد انتخاب شدند. ابزار جمع‌آوری اطلاعات، پرسش‌نامه‌ای با روایی و پایایی قابل قبول بود. پرسش‌نامه شامل اطلاعات دموگرافیک و سازه‌های پاداش درک‌شده، هزینه درک‌شده و نیز بخش تخلفات عمدی پرسش‌نامه رانندگی منچستر بود که به صورت خودگزارشی تکمیل ‌گردید. تجزیه و تحلیل داده‌ها با کمک نرم‌افزار 18SPSS و آزمون‌های آماری t-test، ANOVA ضریب همبستگی و رگرسیون خطی انجام شد. یافته‌ها: آزمودنی‌ها به ترتیب 8/44 و 56/46 درصد نمرات قابل اکتساب پاداش درک‌شده و هزینه درک‌شده را کسب نمودند. مهم‌ترین پاداش درک‌شده رانندگی غیر ایمن، تجربه شور و هیجان بیشتر به دلیل رانندگی با سرعت غیر قانونی (4/56 درصد) و مهم‌ترین هزینه درک‌شده رانندگی ایمن، دیرتر به مقصد رسیدن به دلیل اجتناب از رانندگی به هنگام خستگی (2/51 درصد) عنوان شد. از نظر آماری تفاوت معنی‌داری بین سازه‌های پاداش درک‌شده و هزینه درک‌شده بر حسب جنس، سن، وضعیت تأهل و سابقه تصادفات و جریمه‌ها در طول یک سال گذشته مشاهده گردید (05/0 > P). پاداش درک‌شده و هزینه درک‌شده با یکدیگر و با رانندگی غیر ایمن دارای همبستگی مثبت و معنی‌داری در سطح 01/0 دارند. سازه‌های پاداش و هزینه درک‌شده با یکدیگر، 28 درصد از واریانس رانندگی غیر ایمن را پیش‌بینی می‌کنند که در این میان نقش پاداش درک‌شده (382/0 β =) بیش از هزینه درک‌شده (194/0 β =) می‌باشد. نتیجه‌گیری: پاداش و هزینه درک‌شده نقش مهمی در اتخاذ رفتارهای غیر ایمن هنگام رانندگی دارند. کاهش پاداش و هزینه درک‌شده با هدف افزایش رفتارهای ایمن رانندگی می‌توانند به عنوان یک اصل در امر آموزش و سیاست‌گذاری ایمنی جاده مورد توجه قرار گیرند. به علاوه سازه‌های پاداش و هزینه از هم متمایز هستند و هر کدام می‌توانند به عنوان تعیین‌کننده‌های مستقل رفتار در رانندگی مورد توجه قرار گیرند.

Title: ارتقای سلامت در بيماران مبتلا به سرطان با استفاده از روش PBL

http://hsr.mui.ac.ir/index.php/jhsr/article/view/252

مقدمه: سرطان دسته‌اي از بيماري‌ها می‌باشد كه با رشد كنترل نشده سلولي، تهاجم به بافت‌هاي موضعي و متاستاز سيستميك مشخص مي‌شود. ميزان بروز سرطان طي 50 سال گذشته به طور يكنواخت افزايش یافته است. تغذيه، فعاليت بدني و روحيه اميدوارانه از فاكتورهاي بسيار مهم در بيماري سرطان مي‌باشند كه متأسفانه بيماران آگاهي و نگرش صحيحي در زمينه اين مسایل ندارند. در اين پژوهش به جاي تزريق و انتقال يك سويه مطالب آموزشي، بيماران و خانواده آنان با به كارگيري روش PBL (Problem based learning) به مشاركت فعال در فرايند يادگيري دعوت شدند. اين روش در حقيقت نوعي آماده كردن فراگير براي زندگي است زيرا زندگي، مواجه شدن با مسایل و كوشش براي حل آن‌ها می‌باشد. هدف از اين پژوهش ارتقای آگاهي و تغيير نگرش بيماران و خانواده آنان در خصوص اهميت تغذيه و فعاليت بدني در جريان بيماري و درمان آن می‌باشد. همچنين ارتقای اميد در بيماران با به كارگيري روش يادگيري بر اساس حل مسأله (PBL) مدنظر می‌باشد. روش‌ها: اين مطالعه يك بررسي از نوع شاهددار اتفاقي شده (Randomized controlled trial (RCT)) مي‌باشد. جمع‌آوری داده‌ها از طریق تکمیل پرسش‌نامه سنجش آگاهي و نگرش در قالب مدل بزنف و پرسش‌نامه اميد هرث (Herth hope index (HHI)) قبل و بعد از مداخله در بيماران گروه مورد و شاهد (هر گروه 25 نفر) و همچنین تکمیل پرسش‌نامه سنجش آگاهي و نگرش در خانواده بيمار به عنوان مراقبين اصلي در دو گروه مورد و شاهد (هر كدام 25 نفر) صورت گرفت. مداخله آموزشي و ارتقایي براي بيماران و خانواده گروه مورد به صورت چهره به چهره و انفرادي و با روش PBL انجام شد. داده‌ها با استفاده از نرم‌افزار آماري SPSS14، آزمون‌هاي کای اسکویر (2χ) و آناليز كوواريانس در سطح معني‌داري 05/0 = α مورد تجزيه و تحليل قرار گرفتند. يافته‌ها: ميانگين نمره آگاهي در بيماران گروه مداخله به طور معني‌داري بيش از گروه شاهد است (000/0 > P). همچنين انجام مداخله باعث مطلوب‌تر شدن وضعيت نگرش در گروه مداخله شده است (000/0 > P). ميانگين نمرات نرم‌هاي انتزاعي (12/0 = P) و فاكتورهاي قادرسازي (08/0 = P) دو گروه، اختلاف معني‌داري مشاهده نشد. ميانگين نمرات اميد در بيماران گروه مداخله به طور معني‌داري بيش از گروه شاهد به دست آمد (008/0 > P). ميانگين نمره آگاهي در خانواده بيماران گروه مداخله بيش از گروه شاهد به دست آمد (000/0 > P). همچنين انجام مداخله باعث بهبود وضعيت نگرش در خانواده گروه مداخله شد (000/0 > P). نتيجه‌گيري: مداخلات آموزشي و ارتقایی بر اساس روش PBL در افزايش آگاهي، بهبود نگرش و افزايش اميد بيماران و خانواده آنان مؤثر هستند. بنابرين برای ارتقاي كيفيت زندگي بيماران مبتلا به سرطان، شايسته است ارایه خدمات آموزشي با روش‌هاي مناسبي مانند PBL با مشاركت فعال بيمار و خانواده در فرايند بحث با برنامه‌ريزي مدون انجام پذيرد.

Title: بررسی سبک زندگی ارتقاء دهنده سلامت و رابطه آن با کیفیت زندگی در دانشجویان مقطع کارشناسی دانشکده بهداشت دانشگاه علوم پزشکی اصفهان

http://hsr.mui.ac.ir/index.php/jhsr/article/view/253

مقدمه: سبک زندگی ارتقاء دهنده سلامت جزء معطوف به ارتقای سلامت از سبک زندگی است که دارای شش بعد "فعالیت جسمانی"، "تغذیه"، "مسؤولیت‌پذیری در مقابل سلامت"، "رشد روحی"، "روابط بین فردی" و "مدیریت استرس" می‌باشد. این سبک زندگی علاوه بر تداوم و تقویت سطح سلامت و رفاه باعث احساس رضایت، اقناع شخصی و خود شکوفایی می‌شود. با عنایت به اهمیت چگونگی تأثیر رفتار جدید بر "کیفیت زندگی" به عنوان یک عامل انگیزشی برای شروع رفتار و ادامه آن، این مطالعه به بررسی رابطه و میزان تأثیر این سبک زندگی و ابعاد آن می‌پردازد. مواد و روش‌ها: این مطالعه، یک بررسی توصیفی- تحلیلی با روش سرشماری از دانشجویان مقطع کارشناسی دانشکده بهداشت دانشگاه علوم پزشکی اصفهان در سال 1389 انجام شد. سبک زندگی ارتقاء دهنده سلامت با استفاده از پرسش‌نامه استاندارد Health promotion lifestyle profile2 و کیفیت زندگی با استفاده از ابزار سنجش کیفیت کلی زندگی از نسخه فارسی پرسش‌نامه استاندارد QLQ-C30 که توسط آقای دکتر منتظری و همکاران به فارسی ترجمه و روایی و پایایی آن مورد بررسی و تأیید قرار گرفته است، اندازه‌گیری شد. جهت تجزیه وتحلیل داده‌ها از آزمون‌های آماری توصیفی و استنباطی استفاده گردید. یافته‌ها: میانگین سنی شرکت کنندگان 12/21 سال و 3/54 درصد از آن‌ها خانم بوده، 100 درصد آنان مجرد بودند. از میان شش بعد رفتارهای ارتقاء دهنده سلامت، "رشد روحی" و "مسؤولیت‌پذیری" به ترتیب با میانگین‌های 224/2 ± 01/22 و 31/2 ± 20 بیشترین و "فعالیت جسمانی" با میانگین 9/2 ± 58/17 کمترین امتیاز را به خود اختصاص دادند. كيفيت زندگي عمومی 7/40 درصد از دانشجويان خوب بود و تنها 8/19 درصد از آن‌ها داراي كيفيت زندگي عمومی متوسط بودند. بيشترين فراواني مربوط به كيفيت زندگي مرتبط با سلامت خيلي خوب (6/58 درصد) و كمترین فراواني مربوط به عالي است (9 درصد). در این مطالعه بين وضعيت تغذيه، فعالیت جسماني، مسؤوليت‌پذيري در مورد سلامت خود، روابط بین فردی و رشد روحی و جنس ارتباط آماري معني‌داري مشاهده نشد، اما بين مدیریت استرس و جنس دانشجويان ارتباط آماري معني‌داري وجود داشت (05/0 = P). بين كيفيت زندگي عمومی دانشجويان و وضعیت تغذيه، فعالیت جسماني، مسؤوليت‌پذيري در مورد سلامت خود، مدیریت استرس آن‌ها ارتباط آماري معني‌داري وجود نداشت، اما بین كيفيت زندگي عمومی و رشد روحی دانشجويان ارتباط آماري معني‌دار وجود داشت. بين كيفيت زندگي مرتبط با سلامت و تغذيه، فعالیت جسماني، روابط بین فردی دانشجويان، رشد روحی ارتباط آماري معني‌دار وجود نداشت، اما بين كيفيت زندگي مرتبط با سلامت و مدیریت استرس در دانشجويان ارتباط آماري معني‌دار وجود داشت. نتیجه‌گیری: کیفیت زندگي عمومی اکثریت دانشجويان خوب است و تنها 8/19 درصد از آن‌ها داراي كيفيت زندگي عمومی متوسط بودند. با توجه به میزان فراواني مربوط به كيفيت زندگي مرتبط با سلامت خيلي خوب (6/58 درصد) و این که كمترین میزان فراواني مربوط به عالي به دست آمد (9 درصد)، این امر شاهدی بر کیفیت زندگی مناسب دانشجویان تحت مطالعه می‌باشد. اتخاذ سبک زندگی ارتقاء دهنده سلامت و ابعاد "رشد روحی" و "مدیریت استرس" حداقل در گروه دانشجویان با کیفیت کلی زندگی رابطه معنی‌دار داشتند.

Title: بررسی عوامل پیش‌بینی کننده رفتار دانش‌آموزان دبیرستانی اصفهان در خصوص مصرف فست فود با استفاده از تئوری رفتار برنامه‌ریزی شده

http://hsr.mui.ac.ir/index.php/jhsr/article/view/254

مقدمه: در سال‌های اخیر مصرف فست فود به طور چشمگیری در بین نوجوانان افزایش یافته است به طوری که حدود 30 درصد کودکان و نوجوانان ایالات متحده به طور روزانه فست فود مصرف می‌کنند. مصرف فست فودها با اضافه وزن، رژیم غذایی کم ارزش و مقاومت انسولین در ارتباط می‌باشد. هدف از این مطالعه، بررسی عوامل پیش‌بینی کننده رفتار دانش‌آموزان دبیرستانی اصفهان در خصوص مصرف فست فود با استفاده از تئوری رفتار برنامه‌ریزی شده می‌باشد. روش‌کار: مطالعه به صورت توصیفی تحلیلی و شامل 521 دانش‌آموز دبیرستانی در رنج سنی 15 تا 18 سال است. روش نمونه‌گیری چند مرحله‌ای تصادفی است. در این مطالعه توصیفی از متغیرهای دموگرافیک و متغیرهای مبتنی بر سازه‌های تئوری رفتار برنامه‌ریزی شده، نگرش، هنجارهای انتزاعی، کنترل رفتاری درک‌شده و متغیرهای مستقل دیگر شامل رفتار گذشته و کنترل رفتار واقعی استفاده شد. داده‌های جمع‌آوری شده توسط نرم‌افزار 14SPSS و آزمون‌های توصیفی و استنباطی تجزیه و تحلیل شدند. یافته‌ها: میانگین سنی دانش‌آموزان 28/16 (89/0 = SD) سال به دست آمد. یافته‌های این مطالعه نشان داد که رفتار (مصرف فست فود) با تحصیلات بالای والدین و درآمد بالای خانوار ارتباط مستقیم دارد (01/0 > P). سازه‌های تئوری رفتار برنامه‌ریزی (نگرش، هنجارهای انتزاعی و کنترل رفتاری درک‌شده) با هم توانسته بودند که 7/25 درصد قصد و 6 درصد رفتار را پیش‌بینی کنند. در این میان نقش نگرش (311/0 = β) در پیش‌بینی قصد بیشتر از سایر سازه‌ها بود. نتیجه‌گیری:یافته‌های این مطالعه همانند سایر مطالعات انجام شده نشان داد که تئوری رفتار برنامه‌ریزی شده پیش‌بینی کننده خوبی برای قصد و پیش‌بینی کننده ضعیفی برای رفتار است.

Title: آگاهی و نگرش زنان 45-40 ساله اصفهان نسبت به پدیده یائسگی

http://hsr.mui.ac.ir/index.php/jhsr/article/view/255

يائسگي يكي از مراحل انتقال طبيعي رشد است كه در زندگي هر زني با افزايش سن، روي مي‌دهد. زنان در طي دوران يائسگي، تغييرات جسمي، ذهني، اجتماعي و رواني خاصي را تجربه مي‌كنند. آشنایي با اين تغييرات و درك دلایل آن به زنان كمك مي‌كند تا با آمادگي بيشتري خود را با اين تغييرات تطبيق دهند. آموزش بهداشت يكي از مهم‌ترين راه‌هاي توانمندسازي زنان است. اولین گام در هر آموزشي شناخت، تجزيه و تحليلِ آگاهي و نگرش افراد مطالعه است. هدف اين مطالعه، بررسی آگاهي و نگرش زنان نسبت به پديده يائسگي است.  روش‌کار: اين مطالعه به صورت مقطعي بر روي 400 زن 45-40 ساله غير يائسه و سالم‌‌ تحت پوشش مراكز بهداشتي– درماني شهري انجام شد. نمونه‌ها بر اساس نمونه‌گيري طبقه‌اي، انتخاب شدند. پس از كسب رضايت، وارد مطالعه شدند. ابزار گردآوري داده‌ها، پرسش‌نامه‌اي مشتمل بر سه بخش اطلاعات جمعيت‌شناختي، سؤالاتي جهت سنجش آگاهي و سؤالات بررسي نگرش بود. اطلاعات گردآوري شده با استفاده از نرم‌افزار آماري SPSS و به وسيله آزمون‌هاي همبستگي پيرسون، همبستگی اسپيرمن، آناليز واريانس و t-test مستقل تجزيه و تحليل شدند. یافته‌ها: ميانگين نمره آگاهي افراد تحت مطالعه 76/10 ± 57/63 و ميانگين نمره نگرش آن‌ها 73/12 ± 21/61 بود. در اين مطالعه ميزان آگاهي 8/0 درصد نمونه‌ها ضعيف، 68 درصد متوسط و 5/38 درصد خوب به دست آمد. در ضمن 5/81 درصد زنان نسبت به پديده يائسگي نگرش مثبت داشتند. آزمون همبستگي پيرسون نشان داد كه ميان آگاهي و نگرش زنان تحت مطالعه، ارتباط معني‌داري وجود ندارد. آزمون همبستگي اسپيرمن نشان داد كه آگاهي و نگرش با وضعيت اقتصادي و سطح تحصيلات رابطه معني‌داري دارد. نتیجه‌گیری: فراهم نمودن دسترسي بيشتر به اطلاعات و برقراري حمايت، گام‌هايي ضروري براي ارتقای سلامت زنان در طي سال‌هاي يائسگي مي‌باشد.

Title: رابطه سواد سلامت با وضعیت سلامت عمومی و رفتارهای بهداشتی در سالمندان شهر اصفهان

http://hsr.mui.ac.ir/index.php/jhsr/article/view/256

مقدمه: سواد سلامت را می‌توان به صورت توانایی خواندن، درک کردن و عمل کردن بر اساس توصیه‌های بهداشتی و سلامتی تعریف نمود. افراد با سواد سلامت ناکافی، با احتمال كمتري اطلاعات نوشتاري و گفتاري ارایه شده توسط متخصصان سلامتي را درك می‌کنند و به دستورات داده شده توسط آن‌ها عمل مي‌كنند. همچنین وضعيت سلامتي ضعيف‌تري دارند و هزينه‌هاي پزشكي بيشتري را متحمل مي‌شوند. در نتیجه امروزه سواد سلامت ناکافی به عنوان یک تهدید جهانی به شمار می‌آید. از این رو هدف از این مطالعه، تعیین وضعیت سواد سلامت، تعیین ارتباط سطح سواد سلامت با وضعیت سلامت عمومی و رفتارهای بهداشتی همچون استفاده از خدمات درمانی و همچنین اتخاذ رفتارهای پیشگیرانه در سالمندان شهر اصفهان به عنوان یک گروه آسیب‌پذیر می‌باشد. روش‌ها: این مطالعه از نوع توصیفی تحلیلی است که بر روی 354 نفر سالمند (60 سال به بالا) در شهر اصفهان انجام شد. روش نمونه‌گیری به صورت خوشه‌ای متناسب با حجم بود. جهت گردآوری اطلاعات از پرسش‌نامه سواد سلامت عملکردی بزرگسالان  (TOFHLA، Test of functional health literacy in adults) استفاده شد. در این پژوهش، داده‌ها و اطلاعات به روش پرسشگری درب منازل جمع‌آوری شد. اطلاعات جمع‌آوری شده از طریق نرم افزار 18SPSS و با به کارگیری آزمون‌های آماری من ویتنی، کراسکال والیس و همبستگی اسپیرمن مورد تجزیه و تحلیل قرار گرفت. یافته‌ها: میانگین سنی آزمودنی‌ها 97/6 ± 67 سال بود. حدود 57 درصد از افراد مورد مطالعه مرد و 43 درصد زن بودند. سواد سلامت 6/79 درصد از سالمندان در حد ناکافی، 6/11 درصد مرزی و تنها 8/8 درصد شرکت‌کنندگان از سواد سلامت کافی برخوردار بودند. سطح سواد سلامت ناکافی در افراد با سنین بالاتر (001/0 P <)، میزان تحصیلات کمتر (001/0 P <)، درآمد پایین‌تر (001/0 P <) و زنان (001/0 P =) شایع‌تر بود. همچنین افراد با سطح پایین سواد سلامت بیش از سایرین به پزشک مراجعه می‌کردند (003/0 P =) و سابقه بستری شدن در بیمارستان (01/0 P =) را داشتند. در این مطالعه، ارتباط آماری معنی‌داری بین سطح سواد سلامت و مراجعه به بخش اورژانس در افراد مورد مطالعه دیده نشد. افراد با سطح سواد سلامت بالاتر، وضعیت سلامت عمومی خود را بهتر ارزیابی کردند (001/0 P <). همچنین آن‌ها بیش از سایرین نسبت به انجام آزمایش‌های غربالگری (PSA، Prostate-specific antigen test) جهت غربالگری سرطان پروستات در مردان (001/0 P <) و (FOBT، Fecal occult blood test) جهت غربالگری سرطان کولورکتال (003/0 P =) مبادرت می‌ورزیدند. بین سطح سواد سلامت و انجام ماموگرافی در زنان سالمند ارتباط آماری معنی‌دار دیده نشد. نتیجه‌گیری: به طور کلی این پژوهش سطح سواد سلامت را در سالمندان بسیار ناکافی نشان داد که این خود لزوم توجه بیشتر به امر سواد سلامت را در برنامه‌های ارتقای سلامت می‌رساند. از این رو با تدوین برنامه‌های جامع، ایجاد رسانه‌ها و مواد آموزشی ساده و قابل فهم و همچنین مداخلات آموزشی کارآمد برای افراد با سواد سلامت ناکافی می‌توان گامی مؤثر برای توسعه مهارت‌های سواد سلامت و ارتقای سلامت در جامعه برداشت. در نهایت بدین طریق می‌توان تأثیرات منفی سطح پایین سواد سلامت را در جامعه کاهش داد.

Title: بررسی ارتباط برخی فاکتورهای جمعیت‌شناختی (دموگرافیک) با سن یائسگی طبیعی در زاهدان

http://hsr.mui.ac.ir/index.php/jhsr/article/view/257

مقدمه: با توجه به عوارض جسمي، روحي و رواني يائسگي‌هاي زودرس و ديررس لازم است عوامل مؤثر بر زمان يائسگي به خصوص فاكتورهاي اقتصادي- اجتماعي شناخته شوند. از آن جایی که سلامت خانواده در وهله اول منوط به سلامت مادران است و این پدیده در زنان اتفاق می‌افتد، از این رو هدف مطالعه حاضر تعيين سن يائسگي و فاكتورهاي اجتماعی مرتبط با آن در شهر زاهدان مي‌باشد. روش‌كار: در اين مطالعه مقطعي تعداد 500 زن يائسه شهر زاهدان در سال 86 از 23 مركز بهداشتي درماني و 15 پايگاه بهداشتي انتخاب و بررسي شدند. نمونه‌گيري از كنار مراكز شروع شد و تا تكميل تعداد 13 نمونه مورد نياز در هر مرکز و با در نظر گرفتن معيارهاي ورود به مطالعه ادامه يافت. اطلاعات با استفاده از مصاحبه جمع‌آوری شد و توسط ANOVA و روش تجزيه به مؤلفه‌هاي اصلي (Principal component analysis (PCA)) و در نهایت با آنالیز رگرسیون خطی چند گانه مورد تجزیه و تحلیل قرار گرفت. يافته‌ها: در این مطالعه متوسط سن یائسگی 3/6 ± 3/47 و میانه سن یائسگی 48 سال به دست آمد. متوسط سن یائسگی در زنان تا به حال ازدواج نکرده (6/44 سال، 04/0 P =)، سطح درآمد پایین (3/45 سال، 043/0 P =)، با طبقه اقتصادی- اجتماعی پایین (2/45 سال، 048/0 P =)، مصرف‌کننده دخانیات (8/45 سال، 039/0 P =) به طور معنی‌داری پایین‌تر از سایرین بود. نتيجه‌گيري: علاوه بر نقش ژنتیک و فاکتورهای قاعدگی تولید مثلی، فاکتورهای اقتصادی- اجتماعی نیز می‌توانند در سن یائسگی مؤثر باشند. به دلیل ارتباط این پدیده با بیماری‌ها، اختلالات، عوارض و میزان مرگ و میر زنان، باید به عنوان یک مسأله اپیدمیولوژیک در جوامع، مورد توجه بیشتر قرار گیرد و از زودرس بودن آن تا حد امکان جلوگیری شود.

Title: طراحي و اعتبارسنجي پرسش‌نامه سنجش خستگي بينايي كاربران پايانه‌هاي تصويري

http://hsr.mui.ac.ir/index.php/jhsr/article/view/259

مقدمه: با رشد سريع تكنولوژي، ابزارهاي مرتبط با آن از جمله كامپيوتر، نمايشگرها و پايانه‌هاي تصويري (Visual display terminals یا VDT) به سرعت در حال گسترش هستند. بر اساس تحقيقات، بيشترين شكايات كاربران VDT، ناراحتي‌هاي چشمي گزارش شده است. هدف از اين مطالعه، طراحي ابزاري مناسب جهت سنجش خستگي بينايي كاربران VDT مي‌باشد. موادو روش‌ها: نوع مطالعه مقطعي، بر روي 248 نفر از كاربران VDT در مشاغل مختلف در سال1390 انجام شد. پس از مطالعه كتب، مقالات و پرسش‌نامه‌هاي مشابه، بانك سؤالات تهيه و پرسش‌نامه اوليه تدوين شد. روايي و پايايي پرسش‌نامه با استفاده از شاخص روايي محتوايي (CVI) و ضريب Cronbach’s alpha تأیید گرديد. يك الگوي نظري چهار عاملي در زمينه خستگي چشمي، از مطالعات قبلي، اقتباس و با استفاده از نرم‌افزار Amose16 و به روش تحليل عاملي تأییدي و محاسبه شاخص‌هاي برازش، اعتبار سازه آن بررسي شد. همچنين با استفاده از نرم‌افزار SPSS18 ضريب همبستگي دروني حيطه‌ها محاسبه و جهت بررسي اعتبار ملاك و سطح‌بندي خستگي چشمي، از نتايج اندازه‌گيري هم زمان خستگي چشمي با دستگاه VFM-90.1 و پرسش‌نامه استفاده شد و نقاط برش به وسیله منحني ROC مشخص شدند. يافته‌ها: پرسش‌نامه در 4 حيطه اصلي استرين چشمي (4 سؤال)، اختلال ديد (5 سؤال)، اختلالات سطح چشم (3 سؤال) و مشكلات خارج چشمي (3 سؤال) به تأیید نهايي رسيد. پايايي نهايي پرسش‌نامه برابر 75/0، حداقل شاخص CVI معادل 75/0، شاخص‌هاي برازش مدل 36/0 = RMSEA، 96/0 = GFI، 92/0 = AGFI و ضريب همبستگي نتايج پرسش‌نامه و دستگاه 87/0- به دست آمد و نقاط برش پرسش‌نامه، معادل 65/0 و 36/2 و 88/3 محاسبه شدند. نتيجه‌گيري: پرسش‌نامه خستگي چشمي داراي 15 سؤال تخصصي بوده، از پايايي و روايي بسيار خوبي برخوردار است. اين ابزار با توجه به تعداد به نسبت كم سؤالات و سادگي جملات، به راحتي مي‌تواند ميزان و سطح خستگي چشمي كاربران VDT را ارزيابي کند.

Title: کارایی نظریه عمل منطقی در تعیین علل مرتبط با نمک دریافتی در زنان مراجعه‌کننده به مراکز بهداشتی درمانی شهر یزد

http://hsr.mui.ac.ir/index.php/jhsr/article/view/258

مقدمه: مصرف نمک زیاد با شدت فشارخون سرخرگی و هایپرتروفی بطنی قلب مرتبط است. با توجه به مصرف زیاد نمک در شهر یزد، این مطالعه با هدف شناسایی تعیین‌کننده‌های مصرف نمک مبتنی بر نظریه عمل منطقی در زنان مراجعه‌کننده به مراکز بهداشتی درمانی شهر یزد انجام شد. روش‌ها: این مطالعه از نوع توصیفی- تحلیلی است که بر روی 247 نفر از زنان مراجعه‌کننده به مراکز بهداشتی درمانی شهر یزد انجام شد. نمونه‌ها به روش نمونه‌گیری چند مرحله‌ای انتخاب شدند. ابزار جمع‌آوري داده‌ها پرسش‌نامه بود که بر طبق سازه‌های نظریه عمل منطقی بر روی سه رفتار مرتبط با مصرف نمک طراحی شد. تجزیه و تحلیل داده‌ها پس از ورود به نرم‌افزار آماری 5/11SPSS با استفاده از آزمون‌های آماری Student t-test، آنالیز واریانس یک‌طرفه، χ2و ضریب همبستگی پیرسون، همبستگی اسپیرمن و رگرسیون خطی صورت گرفت. یافته‌ها: میانگین سنی شرکت‌کنندگان 75/6 ± 91/29 سال بود. همبستگی معنی‌دار بین رفتار مصرف کمتر از یک قاشق چای‌‌خوری نمک هنگام پختن غذا و قصد آن (05/0 > P، 132/0 = r)، بین رفتار خودداری از مصرف نمک حین غذا خوردن با قصد آن (05/0 > P، 422/0 = r) و نگرش آن (05/0 > P، 411/0 = r) و بین رفتار خودداری از مصرف غذاهای پر نمک و نگرش (05/0 > P، 169/0 = r) مشاهده شد. در مطالعه حاضر 43 درصد تغییر در قصد مصرف کمتر از یک قاشق چای خوری نمک حین غذا پختن، 37 درصد قصد خودداری از مصرف نمک حین غذا خوردن و 18 درصد قصد خودداری از مصرف غذاهای پر نمک توسط نگرش و هنجار انتزاعی پیش‌بینی شد که نگرش مهم‌ترین جنبه در پیش‌بینی قصد است. نتیجه‌گیری: با توجه به این که نگرش مهم‌ترین پیش‌بینی کننده رفتارهای مرتبط با مصرف نمک است، تدوین برنامه جهت تغییر نگرش جامعه در این زمینه از اهمیت بسزایی برخوردار است..
